# Psychology of Pain

**Published:** 1912-05-15

**Authors:** Walter B. Pillsbury

**Affiliations:** Professor of Psychology, University of Michigan


### PSYCHOLOGY OF PAIN.

BY WALTER B. PILLSBURY, PH. D.
Professor of Psychology, University of Michigan.

(An address delivered before the Dental Society of the Dental Department, University of Michigan.)

The topic of pain presents a large number of aspects. We may talk of the physiological origin of pain, or the effects of pain, or we might talk of the means of avoiding pain.

We must distinguish clearly between two classes of pain: pain as the result of real sensation, and emotional or imaginary pain. Pain as a sensation is the definite pain accompanying the injury of the nerves, much the same as the sensation of light is produced by the impingement of stimuli upon the retina. This kind of pain may be pleasurable or painful; cold stimulates the pain nerves but the sensation is pleasant, or the stimulation from salt spray may be pleasant; thus pain may be pleasurable if in moderation.

The emotion of pain may be aroused by physical means or by thinking. This painful experience does not only come from the part stimulated but from all parts of the body by reflex paths, by extension of reflexes throughout the organism. This reflex accompaniment is really the most unpleasant part of the pain process. It is difficult to breathe during pain. In the emotional state of pain the visceral muscles very frequently contract; there may follow a general weakening of the body mechanism; the flow of the saliva may be stopped or the hair stand on end in severe pain. Thus widely separated physiological processes give rise to the physiological process that we call emotion.

A point most valuable in the psychological treatment of pain is that these emotional accompaniments of the physiological effects come equally easily through imagination apart from physical pain; in fact in most cases, memories and expectations play a larger part in the arousal of these effects than the actual painful stimulation itself. If the subject does not expect pain he does not get it, or, if he gets it, he is not disturbed by it. This expectational pain may be smiled at as of no significance, but it is probable that this peculiar emotional state with its accompanying physiological processes is the large part of pain; you may say that it is imaginary in the start, but in its spread it may lead to all sorts of physiological effects. There are a number of cases on record where men have died as the result of pure expectation. Emperor Charles V had an elaborate rehearsal of his funeral; the realization was so complete that he is said to have died from the effect. It is said that in Copenhagen a number of physicians got control of a criminal who had been condemned to death; they blindfolded the man and told him he was to be bled to death; a slight scratch was made on the body and then water was let fall on the floor to give him the impression be was bleeding. The time was announced when he was to die and it is said he died on the minute. Because of the dripping of the water he dies from the emotional pain, from the wide-spread physiological effects of the situation and his expectation.

These sudden mental shocks from expectation have a serious affect in producing disturbances and they may so interfere with the vital organs as to result in death. To bring out the importance of these emotional or imaginary processes we may refer to the class of neurasthenics or psychasthenics, who have no disease of a physical nature but who have to be treated by the physician. Practically every organic disease may be simulated by these purely mental diseases.

Freud of Vienna asserts that every one of these mental abnormalities that result in disease conditions comes as a result of some shock, some emotional shock, that must have had its beginning in a very early period of the patient's life.

This gives rise to a mental complex that later grows to a dominant place in his mental life, but is detached from his normal consciousness; most of the shocks are of same painful sexual character, an attempted assault has given the patient a shock that is directly opposed to his moral nature. This shock has become excluded from consciousness; the upper consciousness represses it because it is painful. This complex remains latent until later life, then either without further shock or as a result of another shock of any sort it seems to take new life, new experiences group about it, there is a dissociation process, it breaks off from the upper waking consciousness so that it can not be recalled. Loss of control of certain parts of the body may develop large parts of the skin become anesthetic. If you prick the patient with a pin there will be no sensations, there will be no sensation on one half of the body or no sensation on one part of the hand; but later if you hypnotize the patient so that he comes in touch with the lower ideas, he can tell how many times he was touched. The patient may be blind in one eye, or deaf in one or both ears, or have any disturbance whatever; sensations do not come to the upper consciousness with which the person carries on the every-day activities. The paralysis of the vocal cords is an example. These processes can be cured by suggestion. Persons can be bed ridden for years and then when a real stimulus comes, such as the burning of the house, they possess every movement desired. Faith curing and curing by relics are due to suggestions of this sort, and work a cure more or less permanent.

The more modern methods of treating these imagined or mental ailments consist first in determining what the ideas are and then eliminating them. They were earlier frequently discovered by hypnotizing the patient. In hypnosis the detached complexes are reunited with the ordinary consciousness. This method in not very frequently used now as it seems that hypnotism may have disagreeable after-effects, and is not particularly efficacious. Freud's method of psycho-analysis has proved more satisfactory in his own practice. He determines the source of the ideas by questioning the patient. He finds a starting point in the dreams of the patient, since the disturbing complex is more likely to reveal itself in the dreams. He assumes that the painful memory can escape the repressing influence of the waking consciousness by coming while it is off guard in sleep. When once started the questions are continued until the whole complex is discovered. As soon as the patient has fully faced and confessed the incidents that constitute the complex, the trouble disappears. The paralyses are cured, the anesthesias are removed and all the other symptoms cease. Freud calls this the cathartic method. In essence it consists in destroying the force of the obsessing ideas by meeting them squarely face to face.

From our present standpoint, these cases are important as indicating that pains of purely mental origin may have all the injurious effects of real organic diseases, and that these effects may be removed by purely mental operations. Mental cures alone can be really efficacious for these purely mental diseases.

Now to come more definitely to the application of this to your own particular field, you will find that the pain you have to deal with is in a very large number of cases of this imagined sort; it is not all sensational pain but is largely emotional; not an actual stimulation of the nerves, but the fear that the pain is coming; the emotional states are aroused by the expectations. A sensitive person comes to the dental office with the expectation that something very disagreeable is to happen. Anything that comes along serves to discomfort the patient, the discomfort in the greater part comes from expectation rather than from the actual physical operation.

As to means of avoiding these conditions you cannot lay down any absolute rules. The treatment for the removal of the pain is like the treatment for hysteria above. Two general methods may be mentioned, (1) get the patient to face a pain and it will be of no great proportion, or (2) suggest that there is to be no pain. This may be done by some physical method such as the application of cocain, or by any instrumentation you may be partial to, or by assuring the patient that there will be no pain. In the first case the pain disappears as soon as it is felt since it is largely the dread that does the damage in the nervous person; if you can make the patient face it by reasoning with him, he will experience nothing but the physical pain, the emotional pain will be dispelled.

Dubois relates an incident of a physician giving up a practice and going to him in the Alps, he was a strong man but had the idea that he could not work because of failing health, he would be troubled with insomnia or with imagined loss of strength. Dubois suggested to him that there was nothing the matter with him and that he would waste a useful life if he did not attempt to practice. He went back at once, took up his practice and had practically no more trouble.

So on the one hand you can cure pain by having a person face it as pain calling it definitely by this name; if the operator says there will be pain the patient expects it and accepts it.

The other process consists in suggesting there will be no pain. This suggestion may be physical, such as rubbing with an analgesic, they come from your manner, or by distracting attention at the critical moment. When successful, you lose the emotional constituent and get nothing but the physical pain which is much less difficult to bear. There are many variations of these results; twenty years hence you will all have your own peculiar methods of avoiding or relieving pain. Your success in practice will largely depend upon the adequacy of the methods you develop for accomplishing this result. Aside from these two general methods no one can tell you how to attain the end; each man will hit on his little devices and these will most effective for him. Belief in your own method of finding a way will have the same effect upon you as suggestion upon a patient, and if you have success you will have your own favorite drugs, professional mannerisms, etc. More than that, in the way of suggestion there is no possibility of giving you.

Each has to work out for himself his particular method of psycho-therapy.

Physical pain on the other hand has its effect. Shock may be regarded as a reflex of great intensity spreading to much of the nervous system. From the constant slight stimulation of nerves through the degeneration of the tissues there is a wide spread physiological and psychological effect. A man cannot work his best under the effects of pain. The great effect that has been produced in caring for children in school dental clinics has been due to removing irritating conditions. The backwardness of the poorly cared-for children are the definite mental and physical effects of pain. In many cases pain that is not consciously appreciated acts upon the brain to produce a definite physical complex which spreading, leads to mental debility.

My thesis this evening, however, is that imagined pain has almost as great effects as physical pain. You can save your patients from the bad mental effects by proper suggestions just as you can relieve the physical pains by your operative skill. Both together will win you a double debt of gratitude and increased measure of success.

